# The consensus studysquamous cell carcinoma of the oropharynx: late phase clinical trials; core outcomes

**DOI:** 10.1186/1745-6215-14-S1-P78

**Published:** 2013-11-29

**Authors:** Aoife Waters, Catrin Tudur-Smith, Bridget Young, Terry M Jones

**Affiliations:** 1 University of Liverpool, Liverpool, UK; 2 Aintree University Hospitals NHS Foundation Trust, Liverpool, UK

## Background

Contemporary treatments for Oropharyngeal cancer (OPSCC) are associated with a number of debilitating side-effects. Whilst these have a significant impact on the patient's quality of life, they are often not measured in clinical trials of new treatments. In fact, patients and those involved in their care are rarely involved in such outcome selection. As a result, the outcomes chosen are often not relevant to patients or may have little clinical application, and important outcomes may not be measured at all. Furthermore, there is no standardisation of outcome selection and reporting, even amongst trials of comparable interventions. This reduces the data available for meta-analyses leading to difficulties in interpreting a treatment's effectiveness and in making evidence based healthcare decisions. Outcome reporting bias has also been highlighted as a significant problem in the healthcare literature.

## Objectives

To develop a Core Outcome Set for OPSCC clinical trials.

## Methods

A systematic review will identify which outcomes are currently measured in RCTs of interventions for the treatment of OPSCC. Semi-structured qualitative interviews with OPSCC patients and their carers will aim to establish which outcomes such stakeholders think should be measured in OPSCC clinical trials. A Delphi consensus survey, involving patients, carers and clinicians, will identify which outcomes are prioritised by the different stakeholder groups and finally a consensus meeting involving the same major stakeholders will be used to establish which outcomes should be included in the final COS.

## Results

Preliminary results of the systematic review and interviews will be presented.

